# Distributed Integrated Scheduling Algorithm for Identical Two-Workshop Based on the Improved Bipartite Graph

**DOI:** 10.3390/s25247500

**Published:** 2025-12-10

**Authors:** Yingxin Wei, Wei Zhou, Jinghua Zhao, Zhenjiang Tan, Zhiqiang Xie

**Affiliations:** 1 College of Mathematics and Computer, Jilin Normal University, Siping 136000, China; weiyx@mails.jlnu.edu.cn (Y.W.); zjh@jlnu.edu.cn (J.Z.); tanzj@jlnu.edu.cn (Z.T.); 2 School of Computer Science and Technology, Harbin University of Science and Technology, Harbin 150080, China; xiezhiqiang@hrbust.edu.cn

**Keywords:** improved bipartite graph, distributed integrated scheduling algorithm, multi-substring, sensor-enabled manufacturing

## Abstract

To address the issue of further collaboratively optimizing process continuity, time cost, and equipment utilization in identical two-workshop distributed integrated scheduling, an identical two-workshop distributed integrated scheduling algorithm based on the improved bipartite graph (DISA-IBG) is proposed. The method introduces an improved bipartite graph cyclic decomposition strategy that incorporates both the topological characteristics of the process tree and the dynamic resource constraints of the workshops. Based on the resulting substrings, a multi-substring weight scheduling strategy is constructed to achieve a systematic evaluation of substring priorities. Finally, a substring pre-allocation strategy is designed to simulate the scheduling process through virtual allocation, which enables dynamic adjustments to resource allocation schemes during the actual scheduling process. Experimental results demonstrate that the algorithm reduces the total product makespan to 37 h while improving the overall equipment utilization to 67.8%, thereby achieving the synchronous optimization of “shorter processing time and higher equipment efficiency.” This research provides a feasible scheduling framework for intelligent sensor-enabled manufacturing environments and lays the foundation for data-driven collaborative optimization in cyber-physical production systems.

## 1. Introduction

Against the backdrop of intensifying global manufacturing competition and rapidly iterating market demands, the manufacturing phase faces the dual challenges of enhancing efficiency and controlling costs. Simultaneously, as production models rapidly advance towards intelligence and digitalization, the efficient utilization of equipment resources has become a critical pathway to reduce unit manufacturing costs and enhance the core competitiveness of enterprises.

Driven by the combined effects of advancing computer technology and increasingly diversified social application demands, product scheduling methods in the manufacturing industry have undergone a corresponding transformation [1,2,3,4,5,6,7,8,9,10,11]. For example, Reference [1] addresses the production scheduling challenges of sequence-dependent setup and multiple job-order constraints in the household appliance final assembly process, proposing an optimization method that integrates a hybrid genetic algorithm with an iterated greedy strategy. Reference [2] optimizes the integrated scheduling of production and logistics across multiple workshops and proposes a multi-objective artificial bee colony algorithm and corresponding optimization strategy. Reference [3] targets the no-wait flow-shop scheduling problem requiring the simultaneous optimization of makespan and total energy consumption, introducing a hybrid discrete state transition algorithm. Reference [4] focuses on the underestimation of outsourcing value and irrational manual decision-making in manufacturing enterprises, proposing an improved whale optimization algorithm for dynamic scheduling to formulate reasonable outsourcing decisions and maximize outsourcing value. Reference [5] proposes a novel evolutionary algorithm based on two-dimensional array solutions for the distributed no-wait flow-shop scheduling problem.

The current trend toward personalized and diversified demands for complex products has driven the widespread adoption of multi-variety, small-batch production modes. The multi-process machining of such products is closely linked to assembly stages. Although traditional flow-shop and job-shop scheduling methods are effective in large-scale production scheduling, they fragment the intrinsic connection between machining and assembly, making it difficult to achieve efficient coordinated scheduling under complex constraints. Therefore, for complex products with tree-structured constraints, the integrated scheduling method significantly enhances resource allocation efficiency through coordinated decision-making on both processing sequences and assembly workflows [12]. This approach not only shortens the total product manufacturing cycle but also improves the overall equipment utilization rate, thereby achieving superior overall scheduling performance. However, despite these advantages, existing integrated scheduling approaches still struggle to simultaneously balance vertical continuity, horizontal coordination, and migration control.

To solve the above problems, this paper proposes a distributed integrated scheduling algorithm for identical two-workshop based on the improved bipartite graph (DISA-IBG). The main contributions are as follows:

(1) The improved bipartite graph cyclic decomposition strategy is proposed. Based on the theoretical foundation of the improved bipartite graph and combined with the structural features of the process tree, it decomposes the tree into multiple process substrings;
(2) The multi-substring weighting strategy is proposed. This strategy comprehensively considers factors such as substring processing duration, substring priority, and the urgency value of subsequent substrings. Based on these factors, it calculates multi-substring weight values and schedules the process sets in descending order of weight;
(3) The substring pre-allocation strategy is proposed. This involves first pre-allocating process sets to both workshops for virtual scheduling, comparing the processing times in the two workshops, and then selecting the workshop with the shortest processing time for actual allocation. Subsequently, it pre-evaluates whether the next substring to be decomposed can further optimize the processing time in this workshop.

## 2. Related Work

In the field of integrated scheduling for complex products, distributed integrated scheduling research holds significant importance, with studies on identical two-workshop collaborative distributed scheduling attracting considerable attention from scholars and experts [13,14,15,16,17,18]. Currently implemented distributed integrated scheduling algorithms include the quasi-critical-path method [13], balancing-oriented grouping methods [14], neighborhood-reshaping heuristics [15], etc.

The quasi-critical-path method (ACPM), such as the two-workshop integrated scheduling algorithm proposed in Reference [13], typically relies on a three-phase strategy consisting of process sequencing optimization, load-balanced pre-scheduling, and migration control. While effective in identifying key vertical scheduling routes, this class of methods tends to overemphasize vertical progression, resulting in inadequate continuity for non-critical processes and a higher frequency of long-path migrations.

The Balancing-oriented grouping method (EP-ISA), exemplified by Reference [14], aims to achieve dual-objective optimization by first forming process groups to minimize makespan and then allocating these groups to workshops to minimize cross-workshop scheduling. Although these methods enhance horizontal balance, further improvements are still needed in vertical scheduling refinement, parallelism enhancement, and continuous-processing optimization.

The neighborhood-reshaping method (PNR-ISA), such as the two-workshop integrated scheduling method proposed in Reference [15], introduces rendering factors to evaluate local neighborhood characteristics and employ a two-stage structure that combines pre-balancing with dynamic rendering. However, this approach can result in unnecessary process migrations, thereby reducing parallelism and limiting overall scheduling efficiency.

Within the dual-workshop integrated scheduling domain, another notable algorithmic design is presented in Reference [16], which developed a dual-workshop scheduling algorithm combining time-selective and backtracking strategies. The approach utilizes process sequencing and time-selective allocation with a reference-time mechanism to trigger backtracking adjustments when scheduling efficiency declines. While effectively balancing parallel processing and serial operation tightness, the method suffers from computational overhead in large-scale instances and demonstrates limited adaptability to dynamic environments due to its fixed reference-time setting.

In addition to these core integrated scheduling methods, several studies in related domains have provided valuable insights. Reference [19] investigated parallel machine scheduling with workforce constraints, revealing challenges in resource coordination that are equally relevant to the integration of workshops. The work in Reference [20] on quality-driven flow shop scheduling highlights the importance of dynamic quality adaptation, whereas the graph-based reinforcement learning approach in Reference [21] demonstrates both the potential and limitations of intelligent algorithms in production scheduling environments.

To address these challenges comprehensively, our research introduces an improved bipartite graph model that provides a dynamic balance mechanism, effectively coordinating vertical critical path advancement with horizontal load balancing while maintaining flexibility for resource coordination and dynamic adaptation in integrated dual-workshop environments.

## 3. Problem Analysis and Mathematical Modeling

### 3.1. Bipartite Graph

Bipartite graphs represent a special class of graph structures in which vertices can be partitioned into two disjoint subsets. Each edge connects vertices belonging to distinct subsets, and no edge connects vertices within the same subset. Owing to its ability to clearly model the relationships between two distinct categories of elements, this structure finds extensive application in task assignment, resource matching, data clustering, and other domains [22,23,24,25,26,27,28,29,30,31,32].

**Figure 1:**
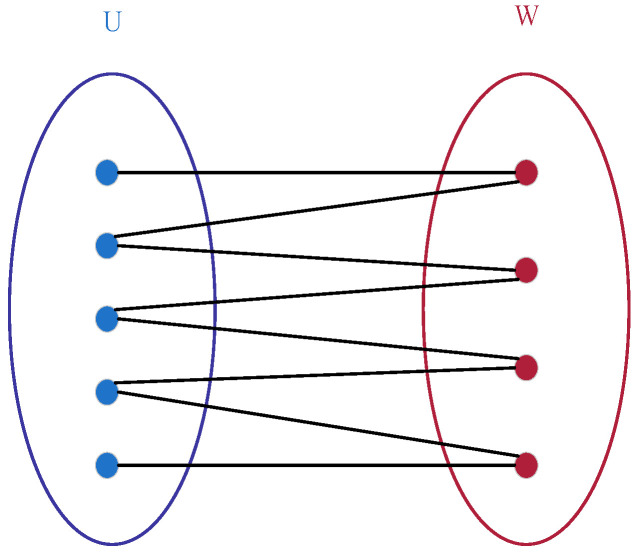
Bipartite graph structure diagram.

Formally, a bipartite graph is defined as an undirected graph G=(U,W,E), where its the vertex set *V* can be partitioned into two disjoint non-empty subsets *U* and *W* (i.e., U∩W=∅ and U∪W=V), and each edge in the graph connects a vertex in *U* to a vertex in *W* (i.e., for any edge e=(u,v)∈E, u∈U and v∈W, or u∈W and v∈U). A schematic diagram of such a bipartite graph is shown in Figure 1.

### 3.2. Integrated Scheduling Analysis

In identical two-workshop integrated scheduling, individual processes are interdependent rather than isolated and are bound by specific constraint relationships that are critical for ensuring schedule rationality and efficiency [12]. Assume that in an identical two-workshop integrated scheduling system, *n* processes are assigned to two workshops, denoted as $\left\{f_{a},f_{b}\right\}$, which collaborate in a specific sequence to complete the product. Each workshop is equipped with *m* machines, and each machine is capable of independently processing every process assigned to the workshop. It is important to note that the two workshops possess identical workshop environments and production conditions, a fact that embodies the core concept of the “identical” property. The specific requirements are as follows:

(1) The processing and assembly operations are uniformly classified into the processing category;
(2) The manufacturing process for complex products can be represented as a tree structure, where each node represents a specific process;
(3) Each process node includes three core parameters: the process number, associated machine number, and processing time;
(4) The equipment resource configuration of identical workshops is completely consistent, and identical equipment is not allowed to be equipped within the same workshop;
(5) Process dependencies exist between processes: a successor process can start only after all its immediate predecessor processes have been completed;
(6) If a process is assigned to a different workshop than its immediate predecessor, then a migration time is incurred;
(7) The makespan of a product is defined as the completion time of the last process;
(8) All time-related quantities—including processing time, start time, migration time, and completion time—are measured in consistent units (man-hours);
(9) The scheduling is strictly non-preemptive: once a process starts on a machine, it continues until completion without interruption.

Accordingly, the mathematical model is established as follows:

(1) $$T=Min\left\{max\left({BT}_{M_{j}A_{i}}+{PT}_{M_{j}A_{i}}\right)\right\}$$

s.t.

(2) $$min{\;BT}_{M_{j}A_{i}}$$

(3) $${BT}_{f,M_{j},A_{i+1}}\geq {BT}_{f,M_{j},A_{i}}+{PT}_{f,M_{j},A_{i}}$$

(4) $${BT}_{{M_{j}A}_{i+1}}\geq {max(BT}_{M_{j}A_{i}}+{PT}_{M_{j}A_{i}})$$

(5) $$min\;\left|\sum_{i\epsilon f_{a}S}{ST}_{i}-\sum_{j\epsilon f_{b}S}{ST}_{j}\right|,\;(f_{a}S\cup f_{b}S=U,f_{a}S\cap f_{b}S=\emptyset )$$

(6) $$min\;M_{f}$$

(1) Indices

*i*: Index of process, representing the *i*th process;

*j*: Index of machine, representing the *j*th machine;

*k*: Index of substring, representing the *k*th substring;

*a*, *b*: Index of workshop, representing workshop *a* or workshop *b*;

(2) Sets

*A*: Process set, $A=\left\{A_{i}\right\},\left(1\leq i\leq n\right)$;

*M*: Machine set, $M=\left\{M_{j}\right\},\left(1\leq j\leq n\right)$;

*S*: Substring set, *S* = {*S_k_*|*k* … {A,B,…,Z};

*f*: Workshop set, $f=\left\{f_{a},f_{b}\right\}$;

$f_{a}S$: Set of substrings assigned to workshop $f_{a}$;

$f_{b}S$: Set of substrings assigned to workshop $f_{b}$;

(3) Parameters

${PT}_{M_{j}A_{i}}$: Processing time required for process $A_{i}$ on machine $M_{j}$;

${PT}_{f,M_{j},A_{i}}$: Processing time of process $A_{i}$ on machine $M_{j}$ in workshop f;

$ST_{i}$: Processing duration of substring *i*;

${ST}_{j}$: Processing duration of substring *j*;

(4) Decision Variables

${BT}_{M_{j}A_{i}}$: Starting time of process $A_{i}$ on machine $M_{j}$;

${BT}_{f,M_{j},A_{i+1}}$: Starting time of process $A_{i+1}$ on machine $M_{j}$ in workshop f;

${BT}_{f,M_{j},A_{i}}$: Starting time of process $A_{i}$ on machine $M_{j}$ in workshop f;

$M_{f}$: Migration time between processes in two workshops.

Formula (1) defines the objective function for the scheduling model, which minimizes the maximum completion time (i.e., the makespan) of the product; Formula (2) requires that each process start as early as possible; Formula (3) represents the sequential processing constraint for processes on the same equipment within the same workshop, meaning that the start time of the successor process cannot be earlier than the completion time of its predecessor on the same machine; Formula (4) ensures that any process can only start after the completion of its immediate predecessor, as formally guaranteed by Appendix A; Formula (5) minimizes the difference in total processing durations of the substrings assigned to the two workshops; Formula (6) minimizes the migration time of processes between the two workshops.

### 3.3. Improved Bipartite Graph

**Figure 2:**
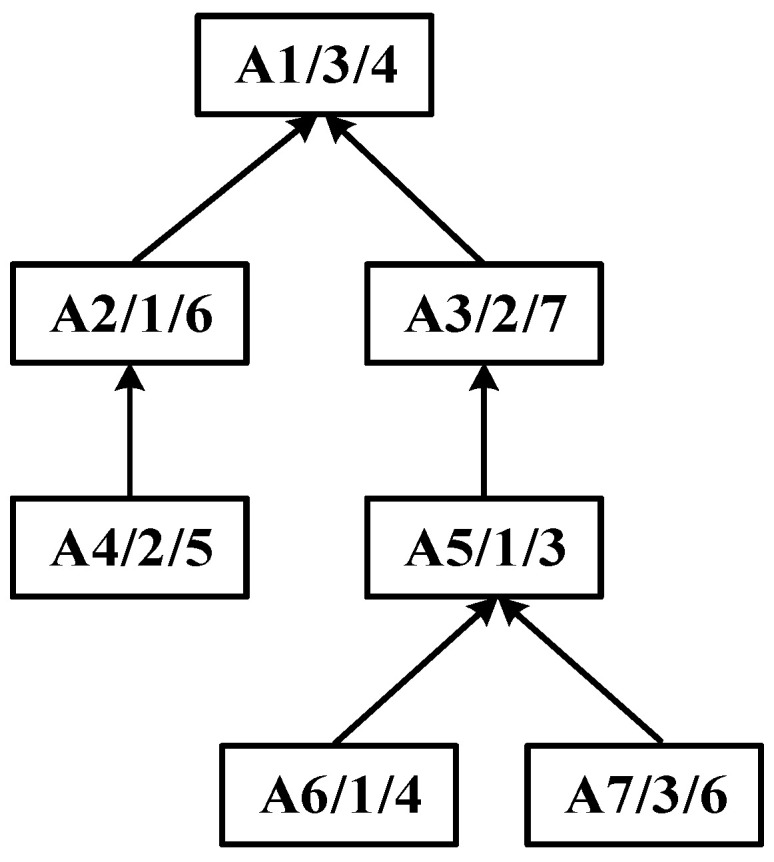
An example of a process tree.

Within the field of integrated scheduling, the manufacturing process is typically represented as a tree-structured graph, referred to as the process tree. Each node in this tree corresponds to a specific process, and each directed edge denotes a precedence constraint. The leaf nodes represent processes that can begin immediately, whereas the root node corresponds to the final process, the completion of which marks the end of the entire product process. Such tree-like structures capture the intrinsic hierarchical dependencies among processes, providing a foundation for constructing the scheduling model. An illustrative example of a typical process tree is shown in Figure 2.

**Figure 3:**
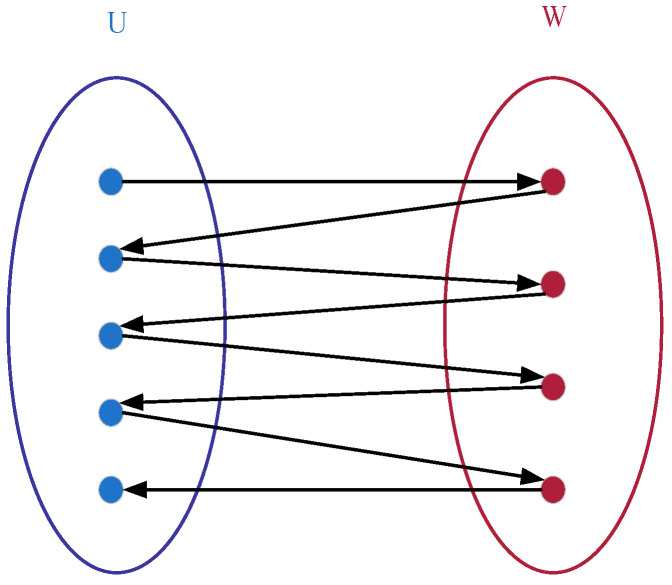
Improved bipartite graph structure diagram with directed edges.

**Figure 4:**
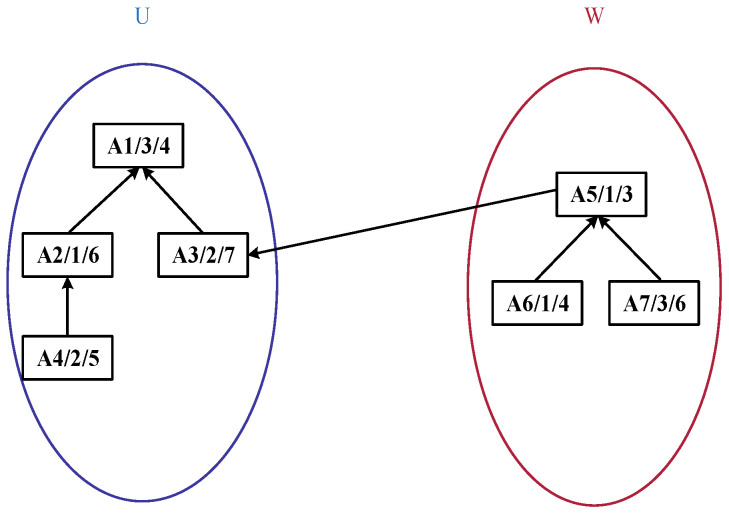
Improved tree-like bipartite graph structure diagram.

Building upon this representation, in the improved bipartite graph, the original undirected graph is enhanced into a directed graph, as illustrated in Figure 3. Vertices within sets *U* and *W* can contain intra-set unidirectional edges, whereas only a single directed relationship is maintained between the two sets. The node positions are further adjusted. The structure diagram after adjustment in accordance with the product process tree structure based on Figure 2 is shown in Figure 4.

In Figure 4, each process node is labeled in the format *A_i_*/*M_j_*/${PT}_{M_{j}A_{i}}$, where *A_i_* denotes the process ID, *M_j_* represents the ID of the machine performing the process, and ${PT}_{M_{j}A_{i}}$ indicates the processing time of the process on the corresponding machine. For instance, the label “A1/3/4” signifies that Process A1 is executed on Machine M3 with a processing time of 4 time units. The arrows in the diagram denote the precedence dependency relationships between processes: an arrow pointing from a preceding process to a subsequent process means the latter can only start after the former is completed (e.g., A4 → A2 implies the execution of A2 depends on the completion of A4). Additionally, the diagram depicts a product process tree, where upper-layer nodes represent assembly or synthesis processes, lower-layer nodes correspond to the sub-processes composing them, and nodes higher up in the tree typically represent process steps closer to the finished product.

### 3.4. Mathematical Modeling of the Improved Bipartite Graph

Let the improved bipartite graph be defined as a quadruple: G=(V,U,W,E), where $V=\{v_{1},v_{2},\ldots ,v_{n}\}$ denotes the finite set of vertices; U⊆V and W⊆V are disjoint subsets; E⊆V×V is a set of directed edges. The detailed mathematical model of the improved bipartite graph is as follows:

(7) V=U∪W,U∩W=∅

(8) $$E=E_{UU}\cup E_{WW}\cup E_{WU}$$

(9) $$E_{UU}=\left\{<u_{k},u_{l}>\in E|\;u_{k},u_{l}\in U\right\}$$

(10) $$E_{WW}=\left\{<w_{m},w_{n}>\in E|{\;w}_{m},w_{n}\in W\right\}$$

(11) $${E_{WU}=1,E}_{WU}=\left\{<Center,u>\in E|\;u\in U\right\}$$

(12) Root=First(U),  Center=First(W)

Equations (7)–(12) collectively define the structural foundation of the improved bipartite graph model. Equation (7) establishes a complete bipartition that strictly divides the vertex set *V* into two mutually exclusive subsets, *U* and *W*. Equation (8) ensures the completeness of the edge set by restricting the edges to three categories: intra-*U*, intra-*W*, and inter-subset directed edge from *W* to *U*. Equations (9) and (10) encode essential precedence relationships by permitting directed edges within subsets *U* and *W*, respectively, thereby capturing both hierarchical and intra-level dependencies. Equation (11) defines a critical structural constraint by specifying that exactly one edge must originate from the first vertex (central node) of subset *W* and point to its immediate successor process in subset *U*. Finally, Equation (12) formally identifies the root node of the process tree as the first vertex in set *U* and the central node as the first vertex in set *W*.

## 4. Algorithm Design and Analysis

### 4.1. Definitions

**Definition** **1.** *Process Chain Duration (PCD). PCD refers to the sum of the standard processing times of all processes within a specific substring. It directly determines the length of the critical path and serves as the core basis for weight calculation*.

**Definition** **2.** *Process Chain Priority (PCP). PCP is determined by calculating the arithmetic mean of the layer priorities of all processes within the substring. This metric quantifies the global influence of the substring in the process tree—the higher the PCP value, the greater the probability that the substring lies on the product assembly critical path, and thus it warrants higher scheduling priority to ensure timely production*.

**Definition** **3.** *Successor Chain Urgency (SCU). SCU represents the number of directly succeeding substrings that remain to be assigned after scheduling the current substring. This metric quantifies the risk of production interruption: the higher the SCU value, the greater the number of successor substrings that would be blocked if the current substring is delayed, thereby indicating that it should be prioritized to avoid production delays*.

**Definition** **4.** *Earliest Finish Time (EFT). EFT is the earliest time at which a process or substring can be completed on its assigned equipment across both workshops, provided that all process constraints are satisfied*.

**Definition** **5.** *Dynamic Center Positioning Strategy (DCPS). In the process tree of a complex product, DCPS assigns sequential labels to all nodes in a top-down, left-to-right order, beginning from the root. Let n denote the total number of labeled nodes (equivalently, the index of the last-labeled node); then, the node indexed ⌊n/2⌋ is defined as the current center node. The center node is dynamically relocated whenever a substring is pruned; after removal, the center of the remaining subtree is recomputed*.

**Definition** **6.** *Substring. In this paper, the term “substring” denotes a minimal schedulable process segment obtained through the cyclic decomposition of the process tree, rather than a string operation. Each substring preserves all precedence relationships of its internal operations and serves as the atomic scheduling unit in the proposed algorithm*.

### 4.2. Performance Evaluation Metrics

To systematically evaluate the performance of substrings in the integrated scheduling framework, a comprehensive set of metrics is employed based on the entropy-weighted TOPSIS methodology. These metrics facilitate objective comparison and prioritization of substrings across multiple scheduling criteria.

#### 4.2.1. Vector Normalization Method

This method is applied in Section 5.2 to eliminate the impact of dimensionality and generate a standardized matrix. The calculation is as follows:

(13) $$z_{ij}=\frac{x_{ij}}{\sqrt{\sum_{i=1}^{n}x_{ij}^{2}}}$$

where $z_{ij}$ denotes the standardized value of the ith evaluation object on the *j*th indicator, $x_{ij}$ represents the corresponding original measured value, *n* is the total number of evaluation objects, and *i* and *j* are the indices of the evaluation objects and the specific indicators, respectively.

#### 4.2.2. Information Entropy Calculation

When applying the entropy weight method to determine the weights, the degree of dispersion for each indicator is first evaluated by its information entropy. The formula is as follows:

(14) $$e_{j}=-\frac{1}{ln\;n}\sum_{i=1}^{n}p_{ij}\;ln\;p_{ij}$$

where $e_{j}$ denotes the information entropy of the *j*th indicator, and $p_{ij}$ is the proportion of the standardized value of the ith evaluation object under the *j*th indicator relative to the sum of all standardized values of that indicator.

#### 4.2.3. Weight Optimization Calculation

Based on the information entropy results, the weights of the indicators are determined. The formula is as follows:

(15) $$w_{j}=\frac{1-e_{j}}{\sum_{j=1}^{m}\left(1-e_{j}\right)}$$

where $w_{j}$ denotes the weight of the *j*th indicator, and *m* represents the total number of indicators.

#### 4.2.4. Weighted Standardized Matrix Calculation

The weighted standardized matrix is then computed by multiplying the standardized matrix by the indicator weights. The formula is as follows:

(16) $$v_{ij}=w_{j}z_{ij}$$

This metric integrates the standardized performance values with the criteria weights.

#### 4.2.5. Ideal Solution Proximity Assessment

To quantify substring performance relative to theoretical optima, two distance-based metrics are employed. The positive ideal solution is denoted as $z^{+}=(z_{1}^{+},z_{2}^{+},z_{3}^{+})$, where each $z_{j}^{+}$ represents the optimal (i.e., most desirable) weighted value of indicator *j* observed across all substrings. Similarly, the negative ideal solution is defined as $z^{-}=(z_{1}^{-},z_{2}^{-},z_{3}^{-})$, where each $z_{j}^{-}$ corresponds to the worst (i.e., minimum) feasible weighted value for indicator *j*. The dimensionality of both $z^{+}$ and $z^{-}$ is three, as the evaluation system incorporates exactly three indicators: PCD, PCP, and SCU—which jointly determine the relative importance of each substring. Accordingly, the upper bound of the summation in Equations (17) and (18) is fixed at 3, reflecting the number of indicators incorporated into the weighted evaluation framework.

Distance to positive ideal solution (optimal values):

(17) $$D_{i}^{+}=\sqrt{\sum_{j=1}^{3}{\left(v_{ij}-z_{j}^{+}\right)}^{2}}$$

Distance to negative ideal solution (worst values):

(18) $$D_{i}^{-}=\sqrt{\sum_{j=1}^{3}{\left(v_{ij}-z_{j}^{-}\right)}^{2}}$$

These two distances jointly capture how close each substring is to the best and worst theoretical performance boundaries, thereby enabling a balanced assessment of their overall processing desirability.

#### 4.2.6. Relative Closeness Coefficient

The relative closeness coefficient measures the aggregate performance of each substring by integrating its distances to both the positive and negative ideal solutions:

(19) $$C_{i}=\frac{D_{i}^{-}}{D_{i}^{+}+D_{i}^{-}}$$

where $C_{i}\in \left[0,1\right]$. A value closer to 1 indicates that the substring is closer to the positive ideal solution, therefore exhibiting a better overall performance.

### 4.3. Theoretical Properties of DISA-IBG

The proposed DISA-IBG algorithm exhibits key theoretical properties that ensure its efficacy in dual-workshop integrated scheduling:

(1) Feasibility preservation. The improved bipartite-graph cyclic decomposition ensures that each generated substring strictly adheres to the precedence constraints of the original process tree. As a result, any resulting schedule inherently maintains global process feasibility across both workshops.
(2) Bounded cross-workshop migrations. The substring pre-allocation strategy ensures that the migration time across workshops is bounded by the number of inter-substring dependencies. In practice, the algorithm further minimizes such migrations by preferentially assigning strongly interdependent substrings to the same workshop.
(3) Continuity-enhancing weighting. By incorporating substring processing duration, priority, and urgency of subsequent substrings, the multi-substring weighting strategy promotes process continuity. Substrings that are longer or time-critical are prioritized, thereby reducing idle intervals and enhancing vertical processing coherence.
(4) Horizontal–vertical coordination. The integration of vertical urgency and horizontal load balancing enables DISA-IBG to effectively reconcile the tradeoff between process-path continuity and resource utilization, mitigating structural biases typical of quasi-critical-path or grouping-centric heuristics.

### 4.4. Improved Bipartite Graph Cycle Decomposition Strategy

The essential property of tree decomposition lies in partitioning the node set of a tree-structured complex graph into several finite and interrelated subsets based on connectivity rules. In this way, the original graph is divided into multiple relatively independent submodules. By solving each subproblem in a distributed manner and integrating the results, the global optimal solution can be effectively approximated [33]. It should be emphasized that this decomposition does not convert the problem into a polynomial-time solvable form; rather, it provides a structured approximation framework for addressing an NP-hard scheduling problem, ensuring tractability while preserving the essential dependency constraints.

**Figure 5:**
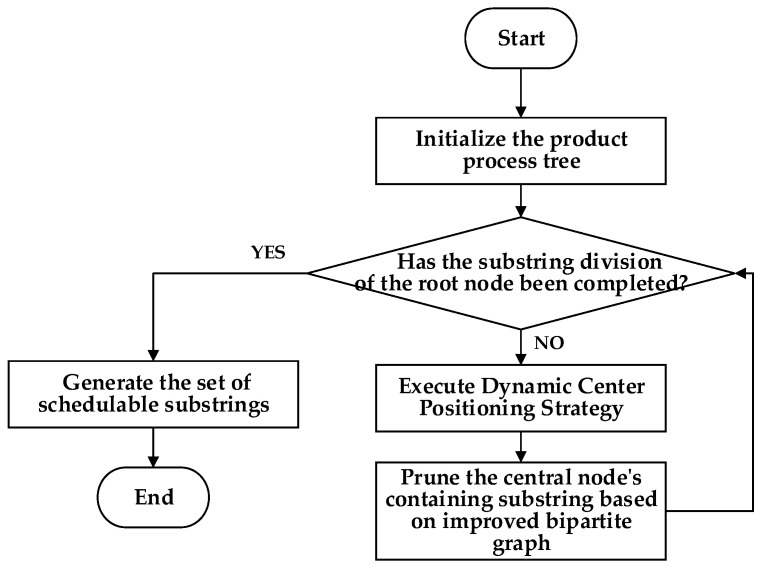
Flowchart of the improved bipartite graph cycle decomposition strategy.

The improved bipartite graph cycle decomposition strategy proposed in this study is centered on achieving recursive and balanced segmentation of the process tree by leveraging the DCPS and improved bipartite graph topological constraints. Specifically, the strategy adopts an iterative recursive framework: in each decomposition round, the current substring nodes are first sequentially renumbered through hierarchical traversal. Then, the position of the center node is determined according to the subgraph size, subject to the following dual criteria: The center node must not only lie at the geometric center of the process tree to ensure balanced subgraph sizes after segmentation, but also be mapped as the first element of the second vertex subset in the improved bipartite graph model, thereby providing an initial matching reference for cross-workshop resource allocation. Once the center node is located, the entire substring associated with its *W*-subset is pruned and designated as an independent scheduling unit, which facilitates subsequent allocation and processing across the two workshops. The remaining part of the process tree is then recursively subjected to center positioning and improved bipartite graph partitioning. Through multiple rounds of iterative refinement, the hierarchical decomposition of the entire process tree is gradually accomplished. This cycle decomposition scheme not only exploits the accuracy of the DCPS in structural recognition but also ensures balanced independence and interdependence of scheduling units through the subset partitioning of the improved bipartite graph. The detailed procedure is shown in Figure 5.

**Figure 6:**
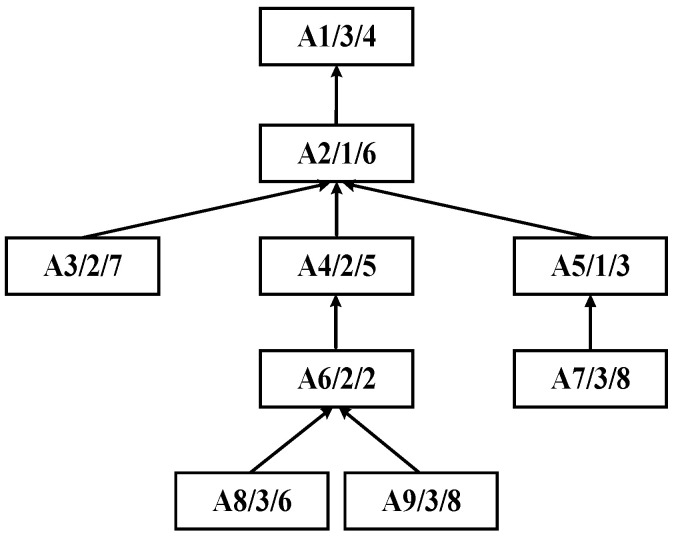
Process tree of complex product A.

Assume that a complex product A consists of ten machining processes $\left\{A_{1},A_{2},\ldots ,A_{10}\right\}$, which are processed on the given machines $M_{1},M_{2},M_{3}$. The corresponding process tree is shown in Figure 6. The following demonstrates the application procedure of the improved bipartite graph cycle decomposition strategy.

**Figure 7:**
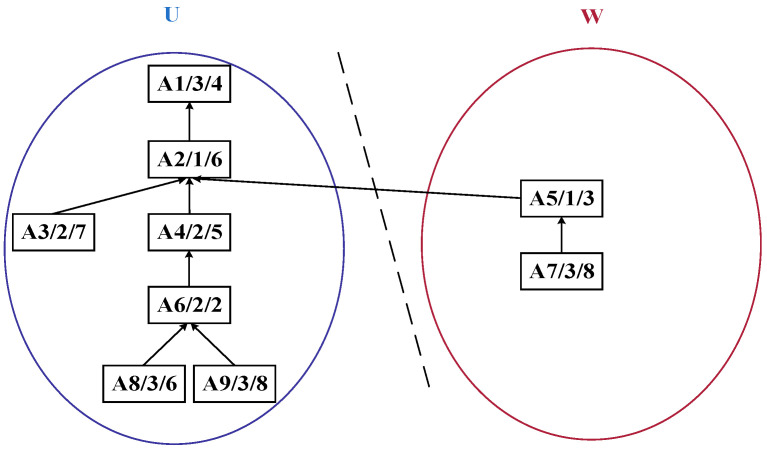
Cut set graph of the process tree of complex product A based on the improved bipartite graph.

**Figure 8:**
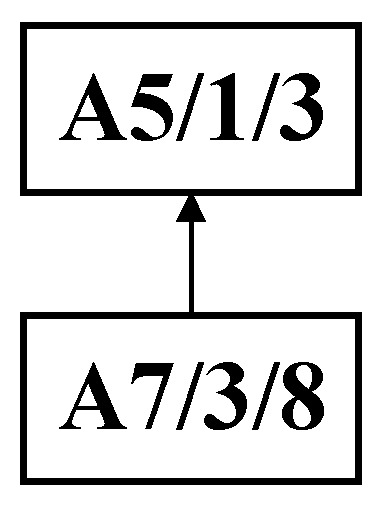
Initial decomposition of complex product A generating process string S_1_.

Step 1: As shown in Figure 7, the DCPS is applied to determine A5 as the center node. Then, the unique connection between the *U*- and *W*-subsets is partitioned, generating the substring {A7, A5}, which forms the process substring S_1_, as illustrated in Figure 8.

**Figure 9:**
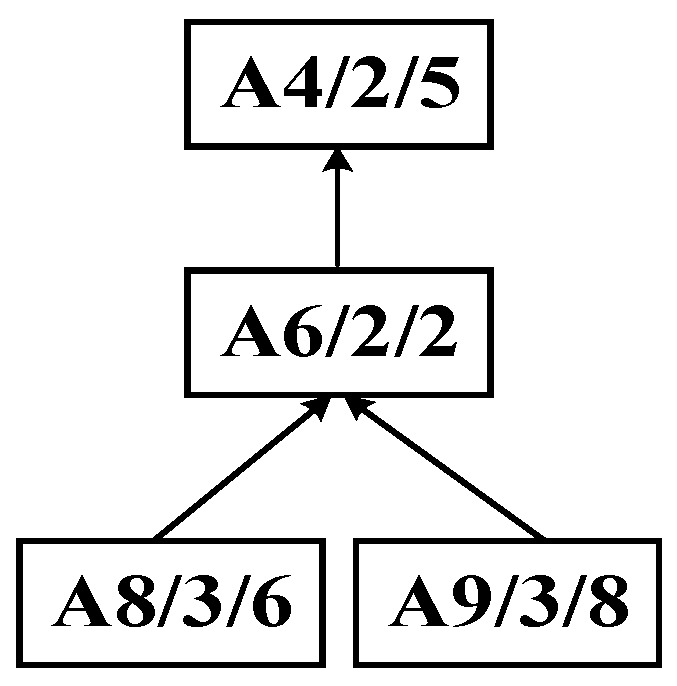
Second decomposition of complex product A generating process substring S_2_.

Step 2: The process tree in the *U*-set obtained from the initial decomposition undergoes a second decomposition using the improved bipartite graph cycle. By applying the dynamic center positioning strategy, process A4 is identified as the center node. Since A4 is the first node in the *W*-set of the improved bipartite graph, the entire substring containing it is pruned, resulting in process substring S_2_, as illustrated in Figure 9.

**Figure 10:**
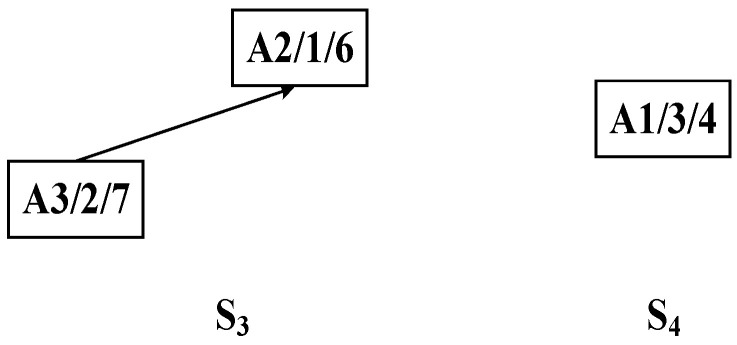
Third decomposition of complex product A generating process substrings S_3_ and S_4_.

Step 3: The process tree in the *U*-set obtained from the second decomposition is further subjected to a third decomposition using the improved bipartite graph cycle decomposition strategy, resulting in process substrings S_3_ and S_4_, as illustrated in Figure 10.

Step 4: According to the predefined cut-set, the process tree of complex product A is recursively decomposed into multiple substring sequences: {A7, A5}, {A8, A9, A6, A4}, {A3, A2}, and {A1}.

### 4.5. Weighted Scheduling Strategy for Multiple Substrings

**Figure 11:**
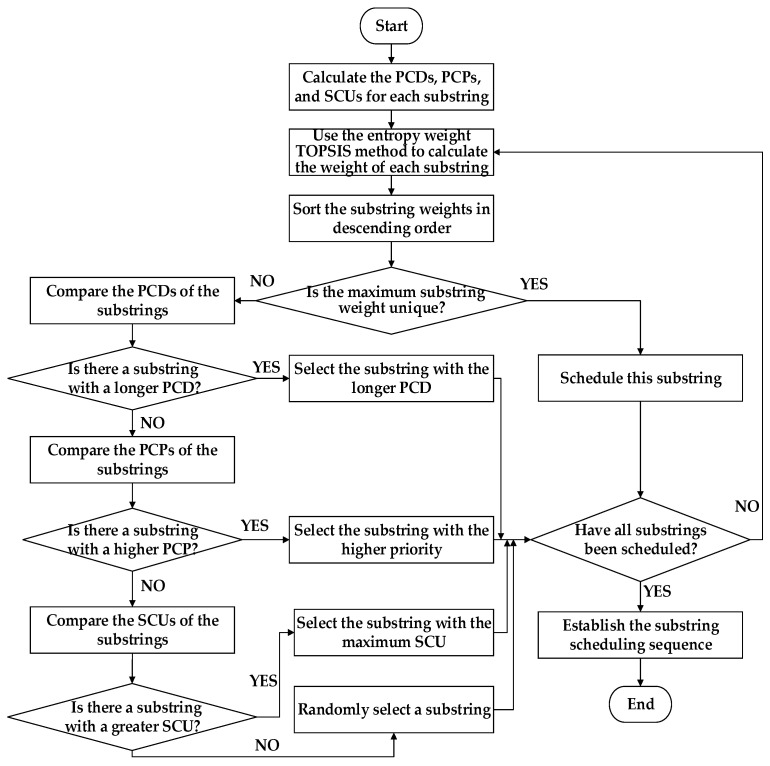
Flowchart of the multi-substring weight scheduling strategy.

In the weighted scheduling strategy for multiple substrings, a priority scheduling sequence of substring process sets is constructed based on the PCD, PCP, and SCU of each substring, enabling efficient scheduling for complex products. Specifically, PCD is measured as the total processing time of all processes within a substring, directly reflecting the continuous occupation of equipment. A longer duration indicates a higher demand for equipment resources. PCP is determined by the hierarchical priorities [34] of the processes in the process tree, ensuring that substrings with higher priority are scheduled earlier. SCU is defined as the number of successor substrings that remain unscheduled after the current substring has been processed. According to the entropy weight method [35], the weights of PCD, PCP, and SCU are calculated to establish an integrated urgency value for each substring process set. The scheduling system then sorts all substrings in descending order of this value and prioritizes the processing of those with higher urgency, thereby effectively reducing equipment idle time resulting from waiting or migration. The flowchart of the weighted scheduling strategy for multiple substrings is presented in Figure 11, with detailed descriptions as follows:

Step 1: Compute the PCD, PCP, and SCU for each substring.

Step 2: Apply the entropy weight method to integrate these parameters and calculate the weight of each substring.

Step 3: Sort all substrings in descending order of weight.

Step 4: Check whether the maximum weight is unique. If so, proceed to Step 5; otherwise, proceed to Step 6.

Step 5: Schedule the substring with the maximum weight and then proceed to Step 10.

Step 6: Compare the PCDs of the substrings.

Step 7: Determine whether there exists a substring with a longer PCD. If so, proceed to Step 8; otherwise, proceed to Step 9.

Step 8: Select the substring with the longest PCD and then proceed to Step 10.

Step 9: Compare the PCPs of the substrings. If a substring with higher PCP exists, schedule it. If PCPs are equal, compare the SCUs and schedule the substring with the larger SCU value. Then proceed to Step 10.

Step 10: Check whether all substrings have been scheduled. If so, proceed to Step 11; otherwise, return to Step 2 to recalculate the weights of the remaining substrings and continue scheduling.

Step 11: Establish the substring scheduling sequence and complete the scheduling process.

### 4.6. Substring Pre-Allocation Strategy

**Figure 12:**
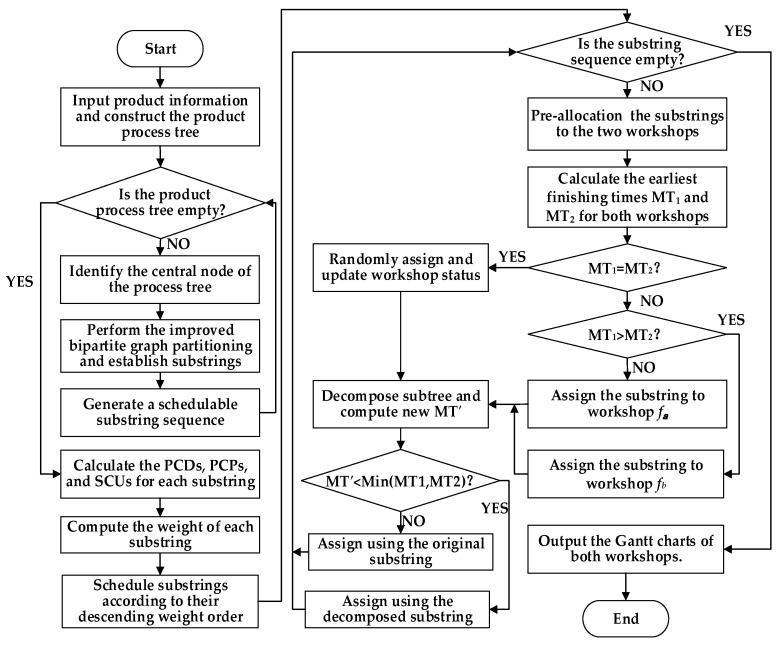
Flowchart of the proposed algorithm.

The strategy first identifies the target substring that needs to be scheduled and preliminarily assigns its process set to the equipment of both workshops. The scheduling time of this process set is then calculated for each workshop, and the one with the shorter processing time is selected as the allocation destination. The original target substring is subsequently decomposed and optimized to further reduce the overall processing time of the product. Through iterative pre-scheduling, structural decomposition, and dynamic allocation, the rationality of resource allocation among substrings is significantly improved. The algorithm flowchart is shown in Figure 12. The specific procedure is described as follows:

Step 1: Select the target substring that currently requires pre-allocation from the set of substrings decomposed from the complex product.

Step 2: Assign the process set of the target substring to workshops $f_{a}$ and $f_{b}$ sequentially for pre-scheduling.

Step 3: Calculate the EFTs of all processes in workshops $f_{a}$ and $f_{b}$ after the pre-scheduling of the substring, denoted as MT_1_ and MT_2_, respectively.

Step 4: Compare MT_1_ and MT_2_. If MT_1_ < MT_2_, select workshop $f_{a}$ as the candidate; if MT_2_ < MT_1_, select workshop $f_{b}$ as the candidate; if MT_1_ = MT_2_, arbitrarily select one workshop as the candidate, and then proceed to Step 5.

Step 5: Perform partial decomposition on the original target substring and calculate the new EFT in the candidate workshop after optimization, denoted as MT′.

Step 6: If MT′ < min (MT_1_, MT_2_), proceed to Step 7; otherwise, proceed to Step 8.

Step 7: Allocate the optimized substring to the candidate workshop and proceed to Step 9.

Step 8: Discard the optimization and allocate the original substring to the candidate workshop, then proceed to Step 9.

Step 9: Check whether all the substrings have been allocated. If so, proceed to Step 10; otherwise, return to Step 1.

Step 10: After substring allocation, update the occupied time slots and available status of the corresponding equipment in the candidate workshop to ensure that subsequent scheduling is aware of resource changes in real time.

Step 11: Output the Gantt charts for both workshops, concluding the allocation process.

### 4.7. Algorithm Complexity

The proposed DISA-IBG algorithm comprises three core computational components: the improved bipartite graph cyclic decomposition strategy, the multi-substring weighting scheduling strategy, and the substring pre-allocation strategy. Among these, the substring pre-allocation strategy dominates the overall computational cost with its $O(n^{2})$ complexity. The formal time complexity of each component is derived as follows.

(1) Improved Bipartite Graph Cyclic Decomposition Strategy

Given a process tree with n processes, the algorithm proceeds by recursively contracting the bipartite graph. In each iteration, identifying admissible edges requires O(n) time, and the problem size is reduced by approximately half. This leads to the recurrence relation: $T_{1}(n)=T_{1}(n/2)+O(n)$.

By the Master Theorem, the time complexity is derived as: $T_{1}(n)=O(n)$.

(2) Multi-Substring Weighting Scheduling Strategy

Given $n_{s}$ substrings (where $n_{s}\leq n$), the entropy-weighted TOPSIS procedure constructs a decision matrix of size $n_{s}\times m$, with m=3 criteria: PCD, PCP, and SCU. Normalization, weight computation, and closeness coefficient evaluation all require linear scans over $n_{s}$ elements, resulting in:$\;T_{2}(n_{s})=O(n_{s}logn_{s})$.

Since $n_{s}=O(n)$, the complexity simplifies to:$\;T_{2}(n)=O(nlog\;n)$.

(3) Substring Pre-Allocation Strategy

The pre-allocation step evaluates inter-workshop migration time and feasibility between all pairs of substrings. This involves constructing and evaluating an $n_{s}\times n_{s}$ adjacency matrix, leading to:$\;T_{3}(n_{s})=O(n_{s}^{2})=O(n^{2})$.

(4) Overall Complexity

The total time complexity is the sum of the three components:$\;T(n)=T_{1}(n)+T_{2}(n)+T_{3}(n)=O(n)+O(nlogn)+O(n^{2})$.

Given the asymptotic relation $n^{2}\gg nlogn\gg n$, the overall complexity is dominated by the substring pre-allocation strategy:$\;T(n)=O(n^{2})$.

## 5. Scheduling Example Analysis

**Figure 13:**
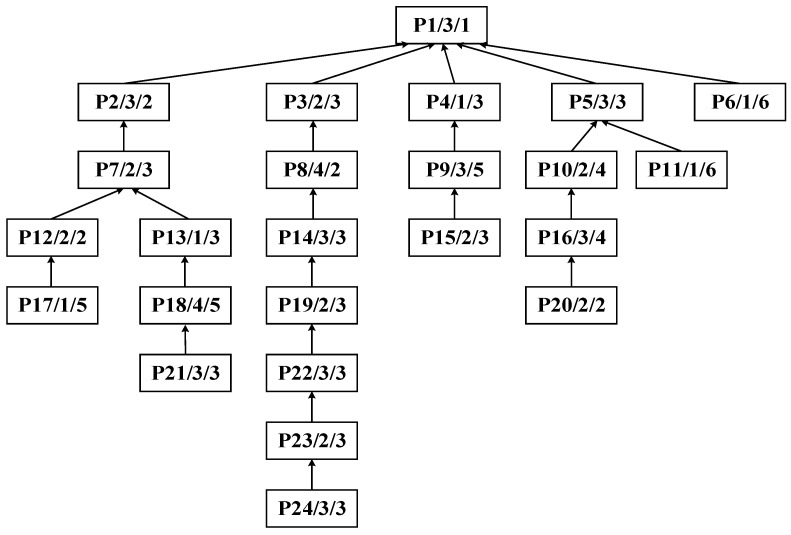
Process tree of complex product P.

The algorithm proposed in this paper exhibits universality and can be flexibly applied to the integrated scheduling research of various complex products. Topologically, symmetric tree structures can be regarded as special cases of asymmetric ones. Therefore, to more comprehensively verify the applicability of the algorithm, a randomly generated asymmetric process tree of complex product P is taken as an illustrative example, as shown in Figure 13. The process tree P comprises 24 processes and 4 machines. The data structure of each process node is a triplet (process ID, corresponding machine, processing time). For instance, node P1/1/1 indicates that process P1 is processed on machine M1 for a duration of 1 h.

### 5.1. Cyclic Decomposition of the Process Tree for Complex Product P

**Figure 14:**
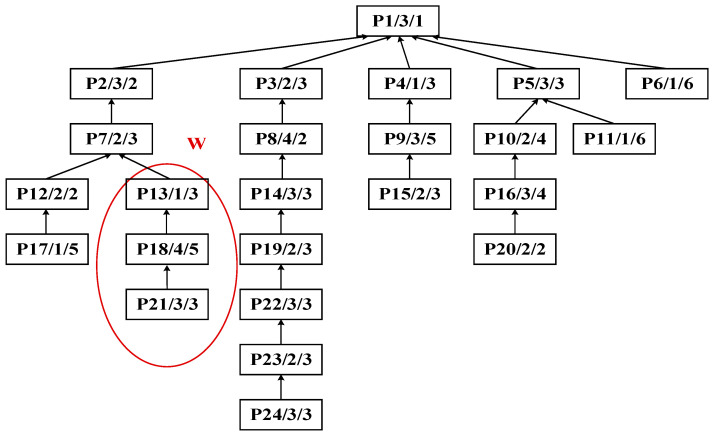
Initial decomposition of the process tree of complex product P.

**Figure 15:**
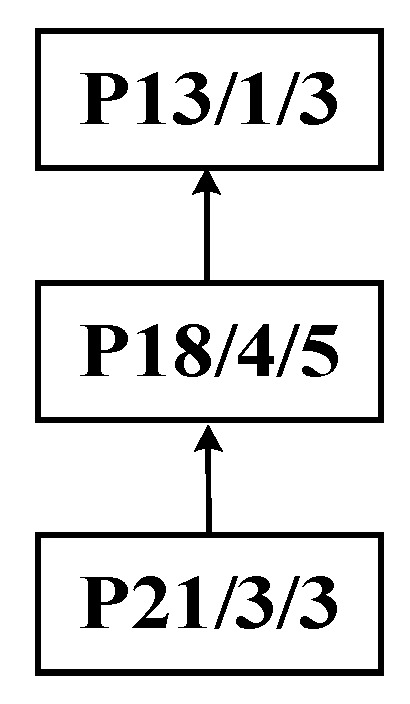
Initial decomposition of complex product P, generating substring S_A_.

Step 1: Identify process P13 as the central node to perform the improved bipartite graph partitioning of the process tree of complex product P, as shown in Figure 14. The initial decomposition yields substring S_A_: {P21, P18, P13} as the cut set, as shown in Figure 15.

**Figure 16:**
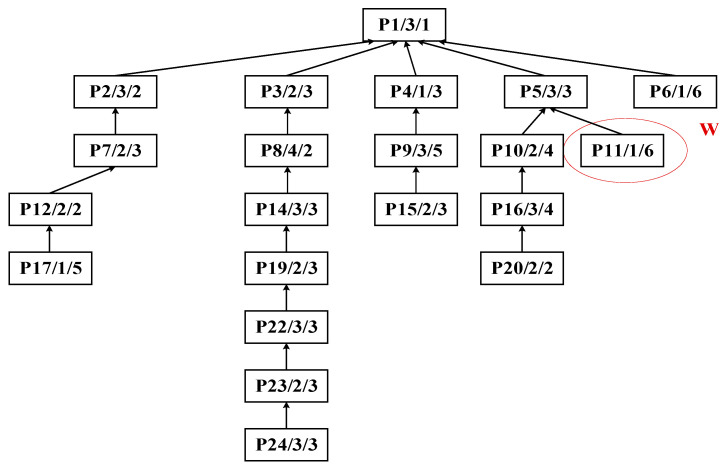
Second decomposition of the process tree of complex product P.

**Figure 17:**
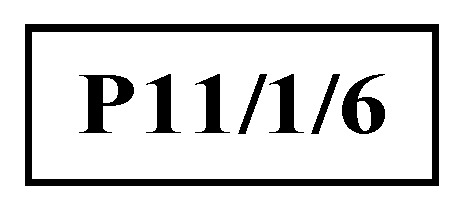
Second decomposition of complex product P, generating substring S_B_.

Step 2: Identify process P11 as the central node for the second partitioning cycle, as shown in Figure 16. This decomposition treats substring S_B_: {P11} as the cut set, with the result illustrated in Figure 17.

**Figure 18:**
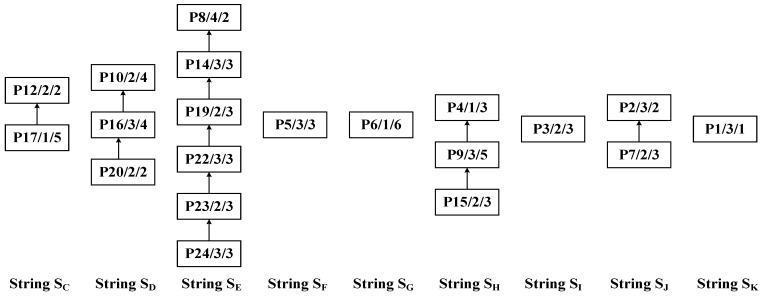
Recursive decomposition of complex product P into substrings S_C_–S_K_.

**Figure 19:**
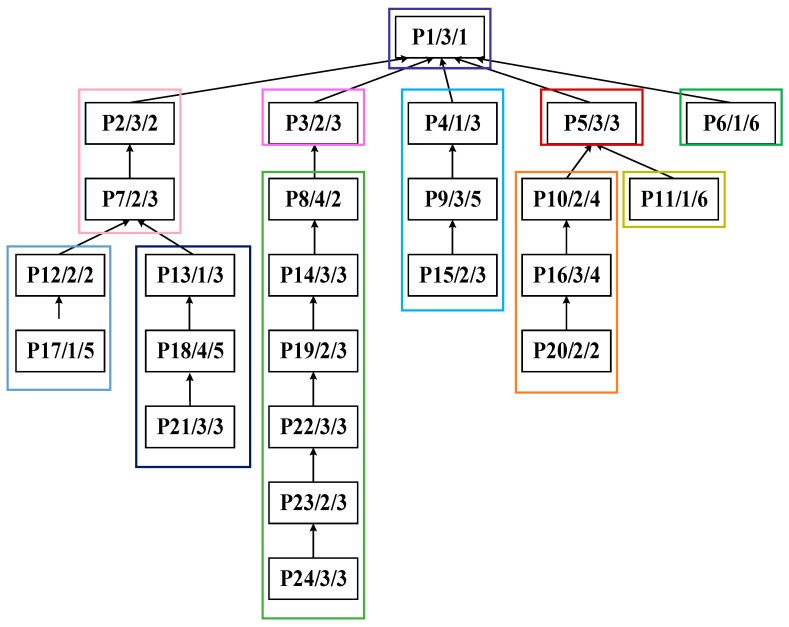
Overall substring-partitioning structure of the process tree of complex product P.

Step 3: Apply the DCPS to sequentially identify processes P12, P10, P8, P5, P6, P4, P9, P2, and P1 as center nodes for further partitioning. The resulting substrings are S_C_: {P17, P12}, S_D_: {P20, P16, P10}, S_E_: {P24, P23, P22, P19, P14, P8}, S_F_: {P19}, S_G_: {P6}, S_H_: {P15, P9, P4}, S_I_: {P3}, S_J_: {P7, P2}, and S_K_: {P1}. The decomposed substrings are shown in Figure 18, and the overall substring partitioning structure is illustrated in Figure 19.

### 5.2. Construction of Scheduling Sequence Based on Multiple Substring Weights

As shown in Figure 19, the 24 processes of complex product P are partitioned into 11 substrings (S_A_–S_K_). The PCDs, PCPs, and SCUs of each substring are calculated, as summarized in Table 1.

The PCD, PCP, and SCU values exhibit differences in units and scales; thus, normalization is required prior to applying the entropy weight method. In this study, the standardization matrix is obtained using vector normalization, as shown in Table 2.

The weights are determined using the entropy weight method, which first computes the information entropy of each indicator and then optimizes them to obtain the final weights. The computed weights for PCD, PCP, and SCU are 38.29%, 22.96%, and 38.75%, respectively. The weighted standardized matrix for each substring is presented in Table 3.

Based on the weighted standardized matrix of substrings obtained above, the ideal solutions can be further determined. Specifically, the positive ideal solution is $Z^{+}=\left(max\;v_{i1},max{\;v}_{i2},max\;v_{i3}\right)=(0.2303,0.1113,0.1550)$, and the negative ideal solution is $Z^{-}=\left(min{\;v}_{i1},min{\;v}_{i2},min\;v_{i3}\right)=(0.0136,0.0202,0.0000)$. Using these solutions, the distances from each substring to $Z^{+}$ and $Z^{-}$ are calculated. The relative closeness coefficient (*C_i_*), which measures the proximity to the ideal solution, is then computed for each substring. The results are summarized in Table 4.

Thus, the final substring scheduling sequence derived from the calculation is S_E_→S_A_→S_D_→S_C_→S_H_→S_B_→S_G_→S_J_→S_F_/S_I_→S_K_.

### 5.3. Allocation Procedure for Dual Workshops

Following the sequencing results obtained in Section 5.2, the substring tasks are progressively allocated to the two workshops. Load balancing is achieved through dynamic adjustments during the allocation process. The detailed allocation procedure is as follows:

Step 1: Based on the weight ranking results, the substring set with the highest weight, S_E_ {P24, P23, P22, P19, P14, P8}, is selected as the initial allocation target and pre-allocated to the machines $\left\{M_{1},M_{2},M_{3},\;M_{4}\right\}$ of the two workshops $\left\{f_{a},f_{b}\right\}$. Since both workshops are initially idle, a random allocation strategy is adopted for the first assignment, with workshop $f_{a}$ being selected. After the allocation, the cumulative processing time of workshop $f_{a}$ is updated to $\sum_{i\epsilon f_{a}S}T_{i}=17$ working hours. The updated scheduling sequence is S_A_→S_D_→S_C_→S_H_→S_B_→S_G_→S_J_→S_F_/S_I_→S_K._

Step 2: For the substring set with the second-highest weight in the current scheduling sequence, S_A_ {P21, P18, P13}, is pre-allocated to the machine sets of the two workshops. Based on the current load of the two workshops ($f_{a}$: 17 working hours, $f_{b}$: 0 working hours), the EFT of substring set S_A_ is calculated by adding its processing time to the current load of each workshop. For $f_{a}$, MT_1_ = 17 + PCD (S_A_); for $f_{b}$, MT_2_ = 0 + PCD (S_A_). Since the EFT in $f_{b}$ is shorter, $f_{b}$ is selected for allocation. Afterward, the cumulative processing time of $f_{b}$ is updated to $\sum_{j\epsilon f_{b}S}T_{j}=11$ working hours.

Step 3: The current scheduling sequence of substrings to be allocated is S_D_→S_C_→S_H_→S_B_→S_G_→S_J_→S_F_/S_I_→S_K_. The first substring set, S_D_, is allocated to the dual workshops. Based on the current cumulative processing times of the workshops ($f_{a}$: 17 working hours, $f_{b}$: 11 working hours), the EFT of S_D_ is calculated: MT_1_ = 17 + PCD (S_D_) for $f_{a}$ and MT_2_ = 11 + PCD (S_D_) for $f_{b}$. The comparison shows that the EFT of $f_{b}$ is shorter; therefore, workshop $f_{b}$ is selected. The indivisibility of substring S_D_ is confirmed through the evaluation of process constraints and temporal optimization, since splitting S_D_ does not reduce its EFT and could potentially disrupt the process sequence. Consequently, the original substring S_D_ is allocated to $f_{b}$, after which the cumulative processing time of $f_{b}$ remains $\sum_{j\epsilon f_{b}S}T_{j}=11$ working hours. The sequence of substrings to be allocated is updated to S_C_→S_H_→S_B_→S_G_→S_J_→S_F_/S_I_→S_K_.

Step 4: Similarly, for the updated sequence S_C_→S_H_→S_B_→S_G_→S_J_→S_F_/S_I_→S_K_, the same iterative allocation logic as in Step 3 is applied. First, the substrings are selected sequentially according to the order of the sequence. Second, based on the real-time cumulative processing times of the two workshops, the EFT of each substring is calculated for $f_{a}$ and $f_{b}$, and the workshop with the shorter EFT is selected. Finally, a feasibility analysis of substring splitting under process constraints is performed—if splitting can reduce the EFT while satisfying process continuity, the substring is allocated in split form; otherwise, it is allocated as a whole.

**Figure 20:**
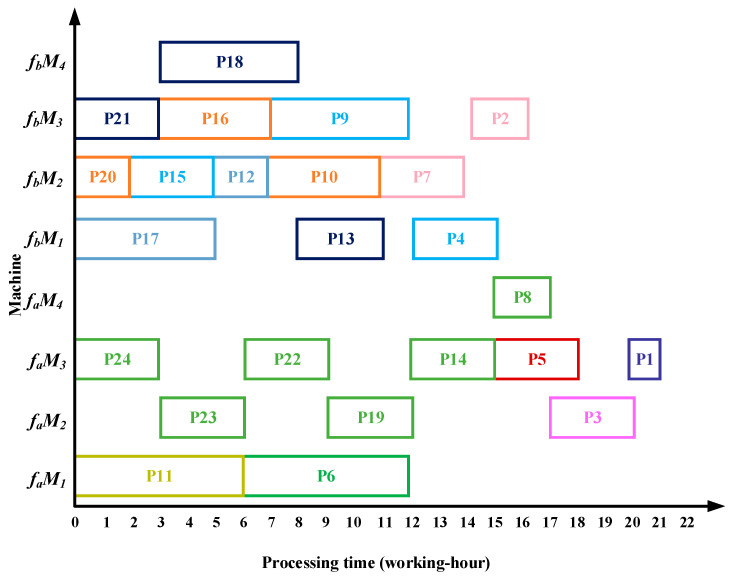
Scheduling Gantt chart obtained by the proposed algorithm.

After all the substrings are allocated, the Gantt chart for processing complex product P in the dual workshops is shown in Figure 20. The total processing time in $f_{a}$ is $\sum_{i\epsilon f_{a}S}T_{i}=21$ $f_{b}$ is $\sum_{j\epsilon f_{b}S}T_{j}=16$ working hours.

### 5.4. Petri Net-Based Sensor Simulation Experiment

Petri nets are modeling tools that integrate graphical representation with formal semantics, offering strong intuitiveness, rich expressiveness, and mature analytical capabilities. Petri net–based modeling and simulation can accurately capture dynamic behaviors, such as concurrency, synchronization, mutual exclusion, and resource contention, while providing deeper insights into the operational patterns of complex systems [36,37,38,39].

**Figure 21:**
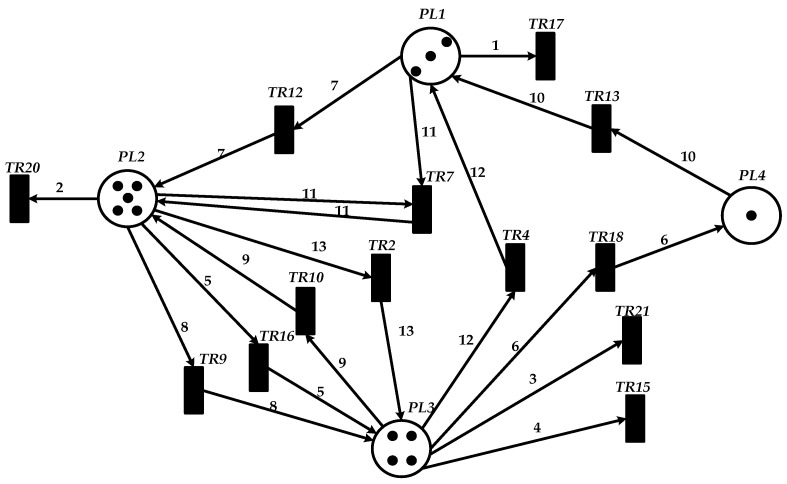
Petri net modeling of product P in Workshop $f_{b}$.

This paper employs the Platform Independent Petri Net Editor V4.3 to construct a Petri net model in Workshop $f_{b}$ for complex product P using a basic Petri net in Figure 21. This model is further extended to a wireless sensor network (WSN) scenario, employing places, transitions, directed arcs, and tokens to characterize state transitions, event triggering, and data transmission mechanisms within the WSN. Places are represented by circular nodes, transitions by short vertical lines, and directed arcs denote the directional relationship from place to transition and from transition to the place. The token values are computed algorithmically.

.

In the Petri net model of Workshop $f_{b}$ shown in Figure 21, places PL1, PL2, PL3, and PL4 correspond to equipment M1, M2, M3, and M4 in the scheduling system of complex product P, respectively. The following transitions represent their corresponding processes: TR2 for P2, TR4 for P4, TR7 for P7, TR9 to TR13 for P9 to P13, TR15 to TR18 for P15 to P18, and TR20 and TR21 for P20 and P21. Specifically, the transitions TR2, TR4, TR7, TR9, TR10, TR12, TR13, TR15–TR18, and TR20–TR21 correspond to processing operations P2, P4, P7, P9, P10, P12, P13, P15–P18, and P20–P21, respectively. Place PL1 is associated with six transitions: TR17, TR13, and TR4, in which it serves as the input place for TR17 and the output place for the remaining transitions. Place PL2 is associated with five transitions: TR20, TR15, TR12, TR10, and TR7, functioning as the input place of TR20 and the output place of the others. Place PL3 corresponds to four transitions, acting as the input place of TR21 and the output place of TR16, TR9, and TR2. Place PL4 corresponds to a single transition and serves as the output place of TR18. The following behavioral relations exist between places and transitions:

(1) Concurrency state: Each independent transition can be enabled in its corresponding place simultaneously. For example, transitions TR20, TR21, and TR19 all hold tokens at time t = 0 and are therefore enabled concurrently in places PL1, PL2, PL3, and PL4, respectively.
(2) Sequential state with strict precedence constraints: A transition can obtain a token and become enabled only after its immediately preceding transition fires and releases the required token to the corresponding place.
(3) Sequential state determined by intra-place ordering constraints: Within the same place, a transition can receive a token only after the preceding transition associated with that place is completed.

The operational state of the Petri net simulation is determined by the distribution of tokens in places. In the experiment, the firing parameter is set to a token count of 13. During the simulation run, after a specific transition is completely fired, the subsequent transition (governed by constraint relations) waits to be triggered, with each transition possessing a definite state and a corresponding time point—thus validating the effectiveness of the simulation experiment.

## 6. Comparative Analysis of Algorithms

All experiments are conducted on a personal workstation configured with the hardware and software specifications summarized in Table 5. The system operates on Windows 11 Professional Edition and is equipped with a 12th Gen Intel(R) Core(TM) i5-12500 CPU (Intel Corporation, Santa Clara, CA, USA), 8.00 GB of RAM, and an integrated Intel(R) UHD Graphics 770 GPU (Intel Corporation, Santa Clara, CA, USA). Multi-instance computational experiments and algorithmic evaluations are carried out using MATLAB R2024b. In addition, Microsoft Visio is utilized for process modeling and diagram construction to ensure clear and standardized graphical representations of system workflows.

### 6.1. Case Results by Four Algorithms

**Figure 22:**
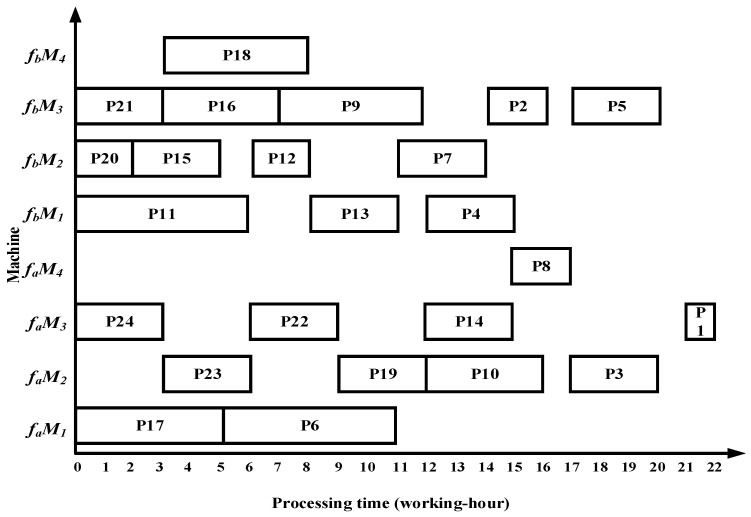
Scheduling Gantt chart by the algorithm in Reference [13].

**Figure 23:**
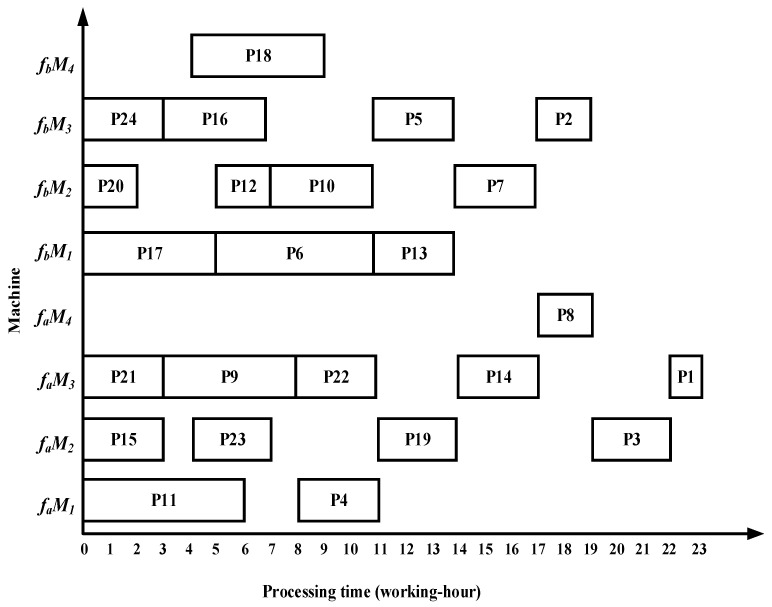
Scheduling Gantt chart by the algorithm in Reference [14].

**Figure 24:**
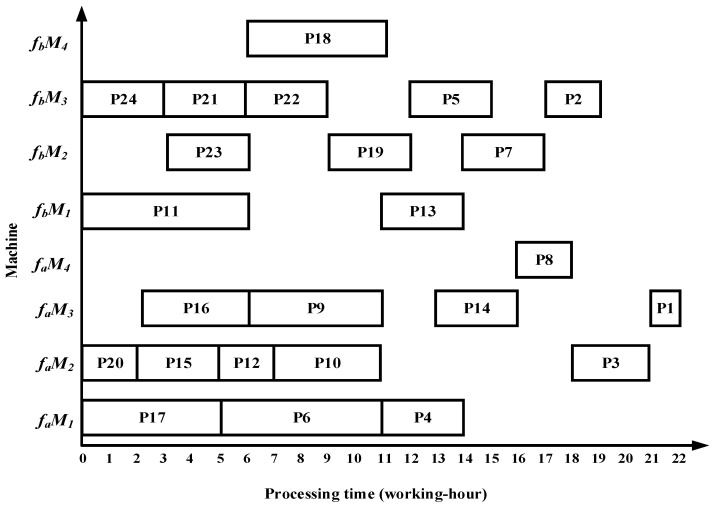
Scheduling Gantt chart by the algorithm in Reference [15].

Taking complex product P as an example, the proposed algorithm demonstrates superior performance over the methods in References [13,14,15] in terms of total processing time, migration time between the two workshops, and equipment utilization. Specifically, the algorithm in Reference [13] requires 22 working hours in workshop $f_{a}$ and 20 working hours in workshop $f_{b}$; Reference [14] requires 23 working hours in $f_{a}$ and 19 working hours in $f_{b}$; and Reference [15] requires 22 working hours in $f_{a}$ and 19 working hours in $f_{b}$. The Gantt charts illustrating the scheduling results of the comparative algorithms are shown in Figure 22, Figure 23 and Figure 24.

The comparative experimental results demonstrate that the proposed algorithm exhibits superior performance compared to the other three algorithms across multiple metrics, including processing time, reduction rate of total processing time, overall equipment utilization, and relative improvement rate of overall equipment utilization, as presented in Table 6.

Compared with the three benchmark algorithms, the proposed algorithm reduces the total processing time by 11.9%, 11.9%, and 9.7%, respectively, while increasing the overall equipment utilization by 2.7%, 5.9%, and 8.7%. Therefore, the proposed method shortens the makespan while enhancing equipment utilization in identical dual-workshop scheduling.

### 6.2. Experimental Results by Four Algorithms

**Figure 25:**
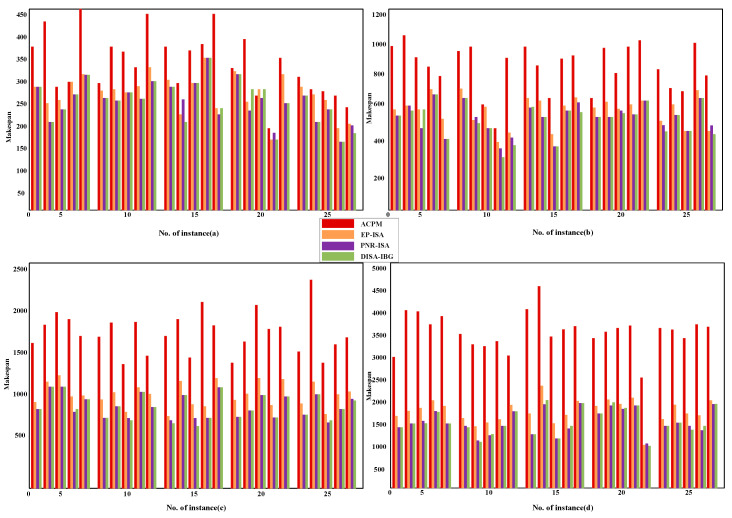
Results of the ACPM, EP-ISA, PNR-ISA and DISA-IBG for 100 instances: (**a**) 20 operations; (**b**) 50 operations; (**c**) 100 operations; (**d**) 200 operations.

To evaluate the adaptability of the DISA-IBG algorithm for products of varying structural complexities in a dual-workshop environment, 100 experimental instances are randomly generated. These instances cover four job scales—20, 50, 100, and 200 operations—with 25 instances for each scale. All scheduling tests are conducted on five machines. Accordingly, the average numbers of operations per machine are 4, 10, 20, and 40, respectively. All product instances are scheduled using four algorithms: ACPM, EP-ISA, PNR-ISA and the proposed DISA-IBG algorithm. The experiments are implemented in MATLAB R2024b on the same PC. Figure 25 presents the makespan results for the 100 instances obtained by the four algorithms. The experimental results demonstrate that DISA-IBG significantly outperforms the other three algorithms across all job scales.

### 6.3. Scalability and Sensitivity Analysis

#### 6.3.1. Scalability Analysis

The proposed DISA-IBG algorithm demonstrates scalability, which stems from two foundational design principles: substring decomposition and distributed decision-making.

(1) Substring decomposition: The improved bipartite-graph framework strategically decomposes the integrated scheduling problem into a set of manageable and largely independent substrings. This decomposition ensures that the computational complexity of resolving each substrings remains constrained and does not scale exponentially with the overall problem size.
(2) Distributed decision-making: The inherent independence of the substrings during the evaluation and weighting phase allows for natural parallelization. This architecture signifies that the algorithm can be efficiently deployed on distributed computing platforms, thereby substantially accelerating solution times for large-scale instances.

Empirical observations corroborate this theoretical scalability. The algorithm maintains stable performance and solution quality as the number of processes scales up to several hundred.

#### 6.3.2. Sensitivity Analysis

Sensitivity analysis is conducted to evaluate the algorithm’s performance stability under variations in key system parameters. The analysis identifies three primary influencing factors:

(1) Process Tree Depth: Increasing process tree depth introduces stricter and more numerous prerequisite constraints. This, in turn, necessitates additional local decomposition operations during the bipartite graph cycle decomposition phase, thereby impacting computational time.
(2) Equipment Consistency: The degree of consistency among workshop equipment significantly impacts scheduling flexibility. Highly identical machine configurations facilitate superior load balancing and minimize scheduling conflicts. Conversely, inconsistent configurations increase matching complexity, potentially affecting the gap in solution optimality.

In summary, the DISA-IBG algorithm demonstrates outstanding overall performance characteristics. It exhibits not only good computational scalability when handling expanding problems but also predictable sensitivity to systematic variations in system parameters. These properties collectively highlight the algorithm’s strong applicability in practical sensor-driven smart manufacturing systems requiring efficiency, resilience, and operational stability.

## 7. Discussion

The superior performance of the proposed algorithm can be mainly attributed to the following reasons.

(1) The algorithm employs an improved bipartite graph cyclic decomposition strategy, which partitions the 24 processes in the process tree of complex product P into 11 substring-level minimal scheduling units. When substrings act as scheduling carriers, their internal processes strictly follow the native topology of the process tree. This methodology not only maintains the precedence constraints between processes within the process tree but also ensures the compact execution of subsequent processes inside each substring. This method thereby effectively avoids the problem of “insufficient continuity of non-critical processes caused by only focusing on vertical critical paths” in Reference [13] and overcomes the defects of “invalid migration and decreased parallelism caused by local rendering” in Reference [15]. For example, compared with the Gantt chart in Figure 22, the proposed algorithm advances process P10, which in turn leads to earlier processing of processes P5 and P1, thereby reducing the total processing time of the dual workshops by 5 working hours. Compared with Figure 23, as processes P23 and P22 are advanced, the subsequent processes P19, P14, P8, P3, and P1 are also advanced, resulting in a total processing time reduction of 5 working hours. Compared with Figure 24, the earlier processing of process P21 leads to the advancement of processes P18, P13, P7, and P2, while the earlier processing of process P14 also advances processes P8, P3, and P1, thereby reducing the total processing time of the dual workshops by 8 working hours.
(2) The proposed algorithm employs a multi-substring weighted scheduling strategy that enhances process execution continuity and compactness. Compared with the lateral or vertical optimization imbalance observed in the other three methods, the proposed algorithm simultaneously considers vertical optimization through PCD and SCU while ensuring horizontal optimization through PCP. Specifically, by incorporating the processing duration of substrings into the decision process, the algorithm effectively avoids a prolonged total processing time caused by scheduling long-duration substrings at the end of the sequence. Meanwhile, by fully accounting for the urgency of subsequent substrings, the algorithm significantly reduces the waiting times between successive processes. This strategy further makes up for the deficiency of “only relying on load balancing and ignoring the vertical urgency of the process” in Reference [14], so that the horizontal equipment load and the vertical process connection can be optimized simultaneously. For instance, compared with Figure 22, Figure 23 and Figure 24, the total processing time in workshop *f_a_* is reduced by 1, 2, and 1 working hours, respectively, while the total processing time in workshop *f_b_* is reduced by 4, 3, and 3 working hours, respectively. Under the proposed method, the overall equipment utilization rate in workshop *f_b_* reaches 83.0%, which represents improvements of 5.8%, 11.8%, and 22.3% over the three comparison algorithms, respectively.
(3) The proposed algorithm employs a substring pre-allocation strategy, which enhances the efficiency of collaborative scheduling between the two workshops and reduces the process migration time between workshops. This strategy avoids the problem of “multiple cross-workshop round-trip migrations leading to a decrease in collaborative efficiency” that is common in References [16,17,18], and improves the overall collaborative efficiency of distributed dual-workshop. For instance, in Figure 22, there are three process migration man-hours: process P17 is processed in workshop $f_{a}$, while its subsequent process P12 is processed in workshop $f_{b}$, forming an inter-workshop migration between P17 ($f_{a}$) and P12 ($f_{b}$). Additional migrations occur between P16 ($f_{a}$) and P10 ($f_{b}$), as well as between P10 ($f_{b}$) and P5 ($f_{a}$). In Figure 23, four process migration man-hours are observed, namely between P21 ($f_{a}$) and P18 ($f_{b}$), P24 ($f_{b}$) and P23 ($f_{a}$), and P11 ($f_{a}$) and P5 ($f_{b}$). In Figure 24, one process migration man-hour occurs between P19 ($f_{a}$) and P14 ($f_{b}$).

## 8. Conclusions

The proposed distributed integrated scheduling algorithm for dual workshops based on the improved bipartite graph takes substrings as the basic scheduling unit and integrates a three-level collaborative mechanism consisting of improved bipartite graph cyclic decomposition, multi-substring weighted scheduling, and substring pre-assignment strategy. This integrated scheduling algorithm enables bidirectional collaborative optimization in both vertical and horizontal dimensions, effectively overcoming the imbalance of “horizontal-priority” or “vertical-priority” issues commonly found in traditional scheduling algorithms.

The proposed algorithm demonstrates significant technological, practical, and environmental benefits. Technologically, it establishes a robust framework for sensor-enabled smart manufacturing, leveraging real-time data for dynamic optimization and ensuring strong operational robustness. Experimental results confirm that the algorithm delivers improved scheduling performance in distributed integrated scheduling of complex products, introduces a new methodological perspective for process-tree scheduling, and contributes to a substantial reduction in the production cycle. Moreover, increased equipment utilization significantly reduces idle energy consumption, offering direct technical support for enterprises to implement low-carbon development concepts, thereby contributing to environmental sustainability.

However, a limitation of this study is its insufficient consideration of the cost-scheduling trade-off within the multi-objective optimization framework. To enhance practical applicability, future work will integrate cost constraints and dynamic disturbance factors, guiding the algorithm toward a more intelligent and robust paradigm for dynamic decision-making.

## Figures and Tables

**Table 1:** PCDs, PCPs, and SCUs of substrings of complex product P.

Substring ID	PCD	PCP	SCU
S_A_	11	5	2
S_B_	6	3	2
S_C_	7	4.5	2
S_D_	10	4	2
S_E_	17	5.5	2
S_F_	3	2	1
S_G_	6	2	1
S_H_	11	3	1
S_I_	3	2	1
S_J_	5	2.5	1
S_K_	1	1	0

**Table 2:** Standardized matrix of substring PCDs, PCPs, and SCUs.

Substring ID	PCD	PCP	SCU
S_A_	0.3891	0.4408	0.4000
S_B_	0.2123	0.2645	0.4000
S_C_	0.2476	0.3967	0.4000
S_D_	0.3537	0.3526	0.4000
S_E_	0.6014	0.4849	0.4000
S_F_	0.1061	0.1763	0.2000
S_G_	0.2123	0.1763	0.2000
S_H_	0.3891	0.2645	0.2000
S_I_	0.1061	0.1763	0.2000
S_J_	0.1769	0.2204	0.2000
S_K_	0.0354	0.0882	0.0000

**Table 3:** Weighted standardized matrix of substrings.

Substring ID	PCD	PCP	SCU
S_A_	0.1490	0.1012	0.1550
S_B_	0.0813	0.0607	0.1550
S_C_	0.0948	0.0910	0.1550
S_D_	0.1355	0.0810	0.1550
S_E_	0.2303	0.1113	0.1550
S_F_	0.0406	0.0405	0.0775
S_G_	0.0813	0.0405	0.0775
S_H_	0.1490	0.0607	0.0775
S_I_	0.0406	0.0405	0.0775
S_J_	0.0677	0.0506	0.0000
S_K_	0.0136	0.0202	0.0000

**Table 4:** Euclidean distances, relative closeness, and scheduling sequence of each substring.

Substring ID	$D_{i}^{+}$	$D_{i}^{-}$	$C_{i}$	Scheduling Sequence
S_E_	0.0000	0.2832	1.0000	1
S_A_	0.0820	0.2131	0.7221	2
S_D_	0.0995	0.1987	0.6664	3
S_C_	0.1370	0.1834	0.5724	4
S_H_	0.1232	0.1500	0.5492	5
S_B_	0.1570	0.1690	0.5184	6
S_G_	0.1820	0.0968	0.3471	7
S_J_	0.1900	0.0926	0.3277	8
S_F_	0.2168	0.0812	0.2724	9
S_I_	0.2168	0.0812	0.2724	9
S_K_	0.2712	0.0000	0.0000	10

**Table 5:** Environment Configuration.

Configuration	Parameter
Operating system	Windows 11 Professional Edition
RAM	8.00 GB
GPU	Intel(R) UHD Graphics 770
CPU	12th Gen Intel(R) Core(TM) i5-12500
MATLAB version	R2024b
Process Modeling Tool	Microsoft Visio Professional 2021

**Table 6:** Comparative analysis of two-workshop scheduling results.

	$\mathrm{Processing}\;\mathrm{Time}\;\mathrm{in}\;\mathrm{Workshop}\;f_{a}$ (Man-Hours)	$\mathrm{Processing}\;\mathrm{Time}\;\mathrm{in}\;\mathrm{Workshop}\;f_{b}$ (Man-Hours)	Total Processing Time (Man-Hours)	Migration Time (Man-Hours)	Reduction in Total Time (%)	Relative Reduction in Migration Time (%)	Equipment Utilization (%)	Relative Improvement in Utilization (%)
Proposed Algorithm	21	16	37	0	----- ^1^	-----	67.8%	-----
Reference [13]	22	20	42	3	11.9%	100%	65.1%	2.7%
Reference [14]	23	19	42	4	11.9%	100%	61.9%	5.9%
Reference [15]	22	19	41	1	9.7%	100%	59.1%	8.7%

^1^ “-----” denotes that the corresponding performance metric is not applicable.

## Data Availability

Data are contained within the article.

## Appendix A

Lemma (Priority Preservation & Deadlock Freedom).
Let the process tree be a DAG T = (A,E).
Let substring decomposition group processes into ordered blocks S = {Pk}, where internal order follows a topological order of T.

Then:

(1) All original precedence relations are preserved under substring scheduling.
(2) The scheduling process cannot generate deadlock.

Proof (Sketch). (Priority preservation)
Since every substring preserves the internal topological order, and substrings themselves are scheduled respecting inter-substring precedence edges, no precedence constraint can be violated.
(Deadlock freedom)
Deadlock requires a cyclic waiting condition among substrings.
However, grouping nodes of a DAG into blocks cannot introduce cycles; thus, the induced substring-level precedence graph remains acyclic. Therefore, at least one substring is always executable (a source node exists), eliminating cyclic waiting.
